# Hippo dictates signaling for cellular homeostasis and immune defense in *Crassostrea hongkongensis* hemocytes

**DOI:** 10.3389/fimmu.2023.1173796

**Published:** 2023-04-24

**Authors:** Fan Mao, Xiaoying Zheng, Nai-Kei Wong, Wenjie Yi, Jingchen Song, Shiwei Fu, Zhiming Xiang, Shu Xiao, Yongbo Bao, Ziniu Yu, Yang Zhang

**Affiliations:** ^1^ Chinese Academy of Science Key Laboratory of Tropical Marine Bio-resources and Ecology and Guangdong Provincial Key Laboratory of Applied Marine Biology, South China Sea Institute of Oceanology, Chinese Academy of Science, Guangzhou, China; ^2^ Southern Marine Science and Engineering Guangdong Laboratory, Guangzhou, China; ^3^ School of Marine Sciences, Ningbo University, Ningbo, China; ^4^ Zhejiang Key Laboratory of Aquatic Germplasm Resources, College of Biological and Environmental Sciences, Zhejiang Wanli University, Ningbo, China; ^5^ Department of Pharmacology, Shantou University Medical College, Shantou, China

**Keywords:** hippo, oyster, innate immunity, signal transduction, hemocyte

## Abstract

**Introduction:**

The Hippo signaling pathway is an evolutionarily conserved signaling cascade that plays a crucial role in regulating cell proliferation, differentiation, and apoptosis. It has been shown to be a key regulator of cell fate and cellular homeostasis in various immune processes. Despite its well-established functions in vertebrate immunity, its roles in marine invertebrate immunity remain poorly understood. Therefore, our present work provides fresh mechanistic insights into how the Hippo pathway orchestrates hemocytic functions in Crassostrea hongkongensis, with implications for studies on its major forms and modifications in animal evolution.

**Method:**

The complete set of Hippo pathway genes, including SAV1, MOB1, LATS, YAP/TAZ, TEAD, and MST, were identified from the C. hongkongensis genome. Quantitative PCR assays were conducted to examine the mRNA expression levels of these genes in different tissues and the levels of these genes in hemocytes before and after bacterial challenges. The study also examined the crosstalk between the Hippo pathway and other immune pathways, such as the AP-1 and p53-dependent p21 signaling cascades. RNA interference was used to knock down MST and TEAD, and MST is a core orchestrator of non-canonical Hippo signaling, to investigate its impact on phagocytosis and bacterial clearance in hemocytes.

**Result:**

The results demonstrated that members of the Hippo pathway were highly expressed in hemocytes, with their expression levels significantly increasing following bacterial challenges. Crosstalk between the Hippo pathway and other immune pathways triggered hemocytic apoptosis, which functioned similarly to the canonical Mst-Lats-Yap signaling pathway in Drosophila and mammals. Knocking down MST resulted in increased phagocytosis and boosted the efficiency of bacterial clearance in hemocytes, presumably due to mobilized antioxidant transcription by Nrf for maintaining immune homeostasis.

**Discussion:**

This study provides novel insights into the regulatory mechanisms underlying the Hippo pathway in immune responses of C. hongkongensis hemocytes. The study highlights the importance of the Hippo pathway in maintaining immune homeostasis and orchestrating hemocytic functions in oysters. Moreover, this study demonstrates the divergence of the Hippo pathway's roles in marine invertebrate immunity from mammalian observations, indicating the need for further comparative studies across species. These findings have significant implications for future research aimed at elucidating the evolutionary trajectory and functional diversity of the Hippo signaling pathway in animal evolution.

## Introduction

First discovered in *Drosophila melanogaster* (1), the Hippo pathway encompasses an array of proteins including the NDR family protein kinase Warts (Wts), WW domain-containing protein Salvador (Sav), Ste20-like protein kinase Hippo (Hpo), adaptor protein Mob as a tumor suppressor (Mats), transcriptional coactivator Yorkie (Yki), and transcription factor Scalloped (Sd) (2, 3). The pathway is evolutionarily conserved, responsible for regulating organ size and maintaining tissue homeostasis in multiple organisms ranging from drosophila to mammals (4). In mammals (5), activation of key kinase Mst1/2 (ortholog of Hpo) results in phosphorylation of Salv1 (the ortholog of Sav) and subsequent activation of Mob1A/B (ortholog of Mats), followed by activation of kinases Lats1/2 (ortholog of Wts). Lats1/2 then recognizes and phosphorylates YAZ/TAZ (the ortholog of Yki), which leads to inhibition of the latter’s ability to cooperate with the TEAD (ortholog of Sd) transcription factor, ultimately inducing apoptosis by downregulating cell growth signaling. Previous works have also shown that knockout of Mst1/2 or overexpression of Yap gave rise to cell proliferation, resistance to apoptosis, and massive organ overgrowth (6–9). These findings suggest that the primary function of the canonical Hippo signaling pathway is to inhibit Yap activation and eventually regulate organ size by restricting cell proliferation while promoting differentiation.

Recently, it has been proposed that Hippo signaling proteins also play pivotal roles in both innate and adaptive immunity (10). The core kinases Mst1/2 initiate non-canonical Hippo pathways, in manners unlike the canonical Mst1/2-Lats1/2-Yap Hippo signaling cassette (11). For example, signaling along the Mst1/2-Mob1 axis is crucial to T cell migration and activation, while Lats1/2 and YAP may not be involved due to their relative insensitivity to Mst1 elimination (12). In the context of regulation of innate immunity by the Hippo pathway, its crosstalk with other pathways has been frequently reported. For instance, Mst1 and Mst2 can be activated *via* a MyD88-dependent pathway, while depletion of Mst1/2 enhances the phosphorylation levels of IκB kinase (IKKα/β), as well as the phosphorylation levels of IκBα (13, 14). Meanwhile, phagocytic oxidative bursts generate large amounts of reactive oxygen species (ROS) that crucially mediate bacterial killing. This innate immune function is considerably impaired in Mst1/2-deficient phagocytes during bacterial infection (15). Overall, Mst1/2 promotes activation of the guanoside cyclase Rac and recruits mitochondria to phagosomes through TLR-TRAF6 signaling cassette following stimulation by bacteria or TLR ligands, which positively regulates the generation of mitochondrial ROS to enhance bacterial in macrophages (14, 16).

Thanks to advances in genome sequencing, knowledge on the Hippo pathway across distant phylae has greatly expanded, further illustrating conservation of the Hippo pathway. Even in the sea anemone *Nematostella*, most of the components present in humans can be identified (17). Evidence suggests that major domains of YAP, especially the WW and TEAD-binding domains, are conserved between cnidarians and mammals (17). Furthermore, YAP and its binding partner TEAD have been observed to co-evolve. While previous studies have clarified the pathway in different evolutionary statuses and provided insights into its function and mode of operation, the nature of the pathway in other protostome groups such as mollusks remain poorly understood. Some recent works have shown that the Hippo pathway may function in response to environmental stressors in mollusks. For example, in the freshwater snail *Lymnaea stagnalis*, exposure to cadmium resulted in upregulation of Hippo pathway genes, suggesting a possible involvement in the snail’s response to heavy metal toxicity (18).

To date, several oyster genomes have been sequenced, among which, *Crassostrea hongkongensis* (the Hong Kong oyster) is one of the most valuable marine invertebrate species cultivated along the coast of South China. It is essential to achieve a better understanding of its immune system to prevent disease and subsequent loss in coastal oyster farms. Recently, our study on LHX9 (a gene highly expressed in oyster hemocytes, which participates in oyster immunity) revealed that depletion of LHX9 resulted in activation of the Hippo pathway (19). The findings inspired us to examine the immune regulation of hippo pathway in oysters in greater detail. Here, we analyzed the full members of canonical Hippo pathway in oyster, and further explored their cross-talk with different pathways. Non-canonical Hippo pathway was also investigated *via* RNA interference, in the hope of shedding new light on their regulatory mechanisms in marine invertebrates.

## Results

### Conservation of core components of the Hippo signaling pathway in representative species

**Figure 1:**
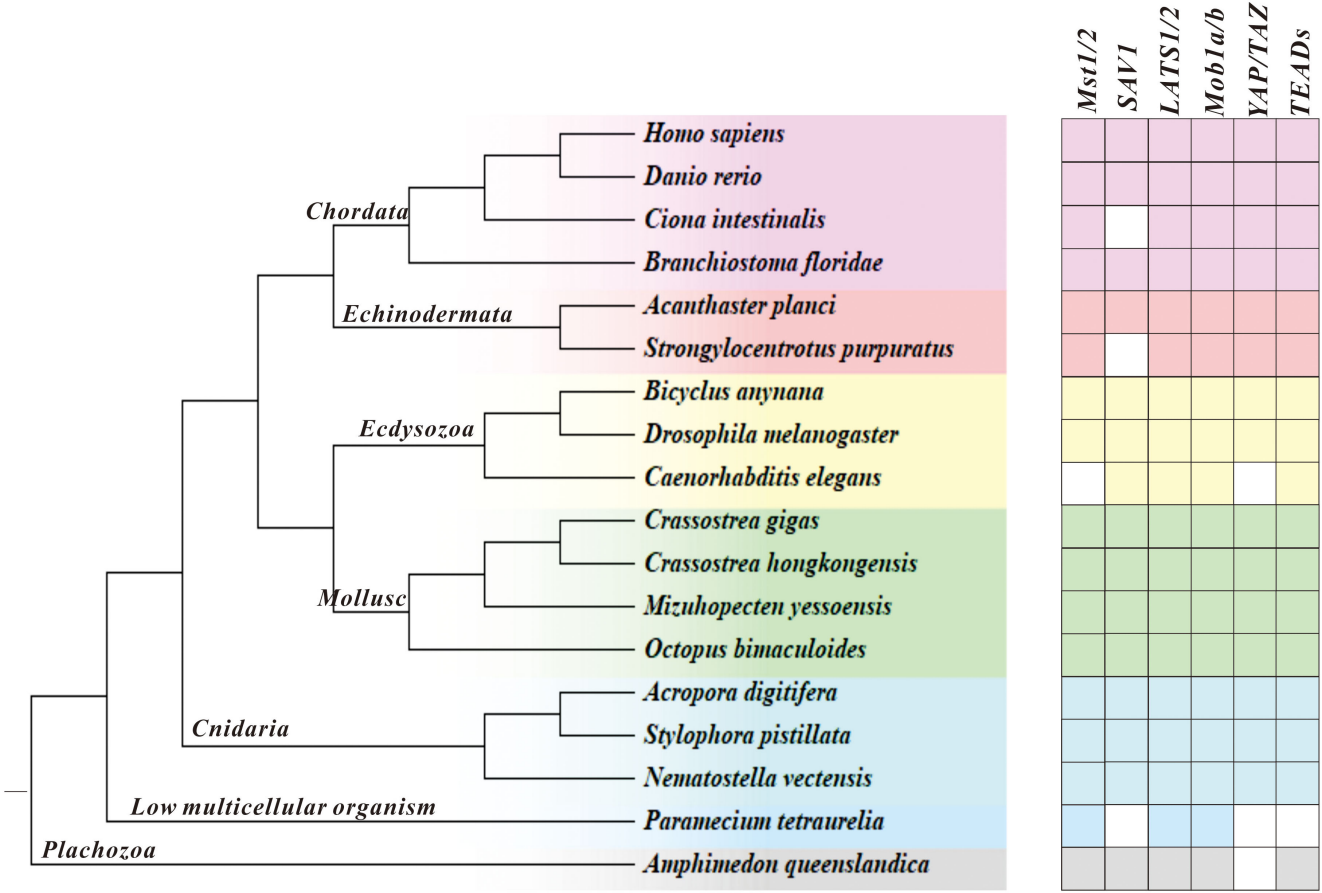
Illustration of the Hippo pathway genes in species ranging from *A. queenslandica* to *H. sapiens*. Blank represents genes that are not detected, and a pentagram marks *C. hongkongensis*, which we studied here.

In this study, we investigated the evolution of the Hippo/YAP pathway in species ranging from *Amphimedon queenslandica* to *Homo sapiens* by using bioinformatics tools. Our results show that, broadly speaking, the Hippo signaling pathway is well conserved during evolution. However, we did observe some genes that appear not identifiable or lost in certain species, as shown in **Figure 1**. Despite this, core components of the Hippo pathway were found to be conserved in Molluscs, including bivalves (*C. gigas*, *C. hongkongensis*, and *Mizuhopecten yessoensis*) and cephalopods (*Octopus bimaculoides*), which suggests a good degree of pathway integrity.

**Figure 2:**
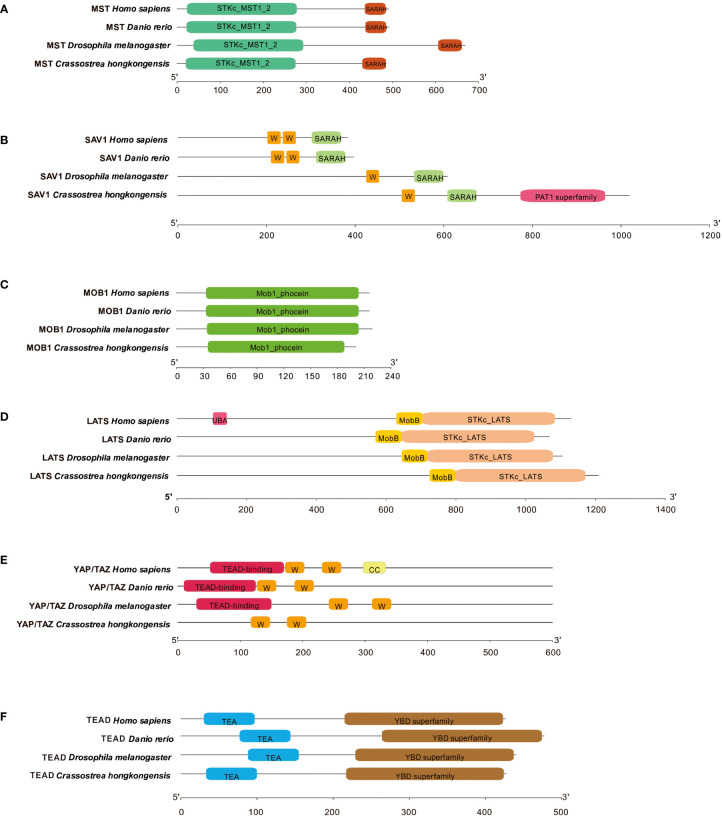
Domain characteristics of ChMST **(A)**, ChSAV1 **(B)**, ChMOB1 **(C)**, ChLATS **(D)**, ChYAP/TAZ **(E)** and ChTEAD **(F)**.

Subsequently, we conducted an in-depth analysis of the protein sequences and domains of *Ch*MST, *Ch*SAV1, *Ch*MOB1, *Ch*LATS, *Ch*YAP/TAZ, and *Ch*TEAD to better understand the Hippo signaling pathway in *C. hongkongensis*. We utilized SMART to identify and compare the conserved domains of these proteins with those found in humans and zebrafish. The analysis reveals that *Ch*MST (**Figure 2A**), *Ch*MOB1 (**Figure 2C**), *Ch*LATS (**Figure 2D**), and *Ch*TEAD (**Figure 2F**) share highly conserved domains with their counterparts in humans and zebrafish. However, *Ch*SAV1 only possesses one WW domain and a SARAH domain for MST binding, in addition to patatin (PAT) domains (**Figure 2B**). Furthermore, the TEAD binding domain of *Ch*YAP/TAZ was found to be not conserved (**Figure 2E**), which may be attributed to sequence divergence or low sequence homology.

### Expression profile of core components of the Hippo pathway in *C. hongkongensis*

**Figure 3:**
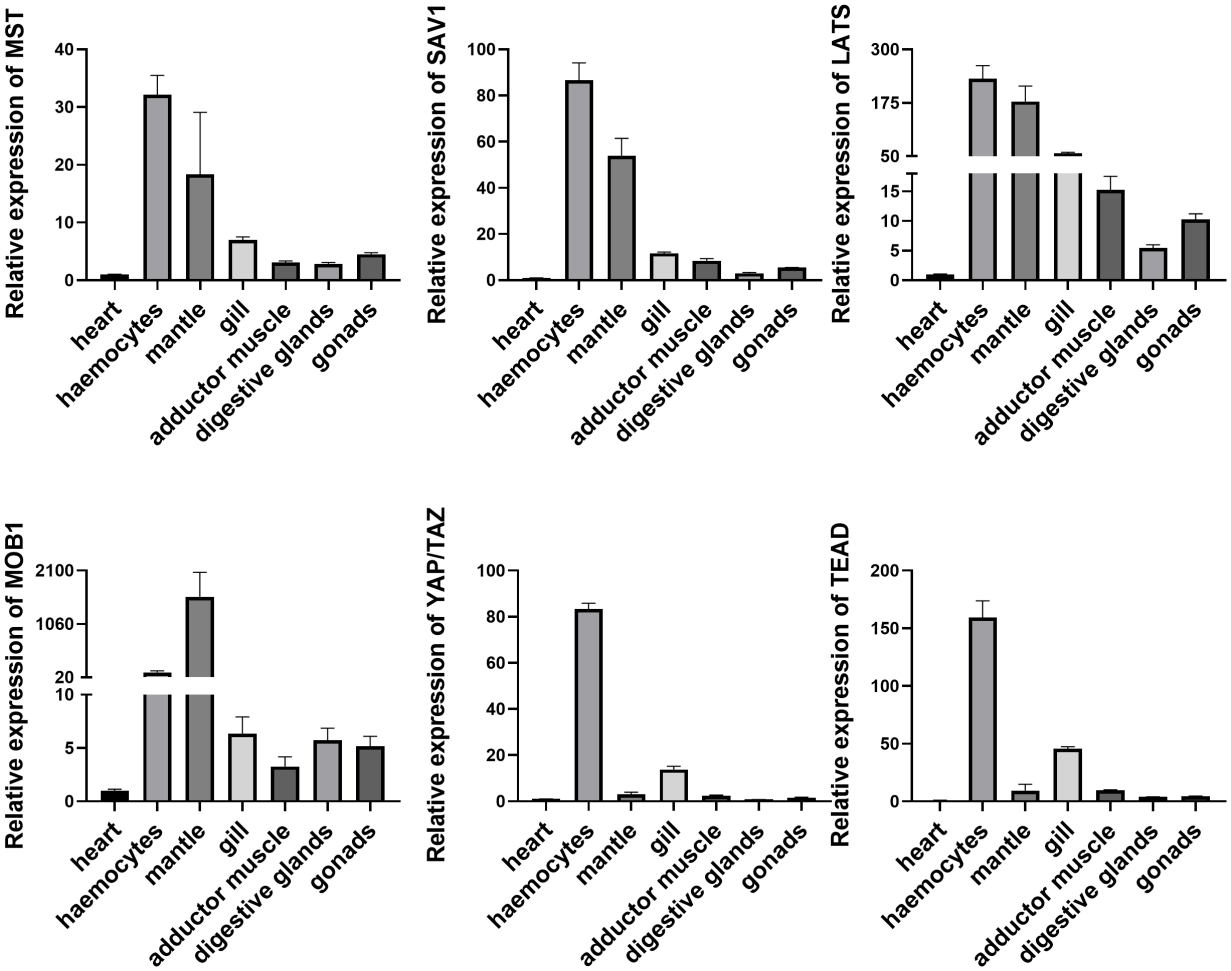
Expression profile of Hippo pathway in different tissues. Seven tissues including heart, hemocytes, mantle, gill, adductor muscle, digestive glands, and gonads were examined here. Data analysis was performed by using GraphPad Prism 9 software, and vertical bars represent mean ± SD (*n* = 3).

**Figure 4:**
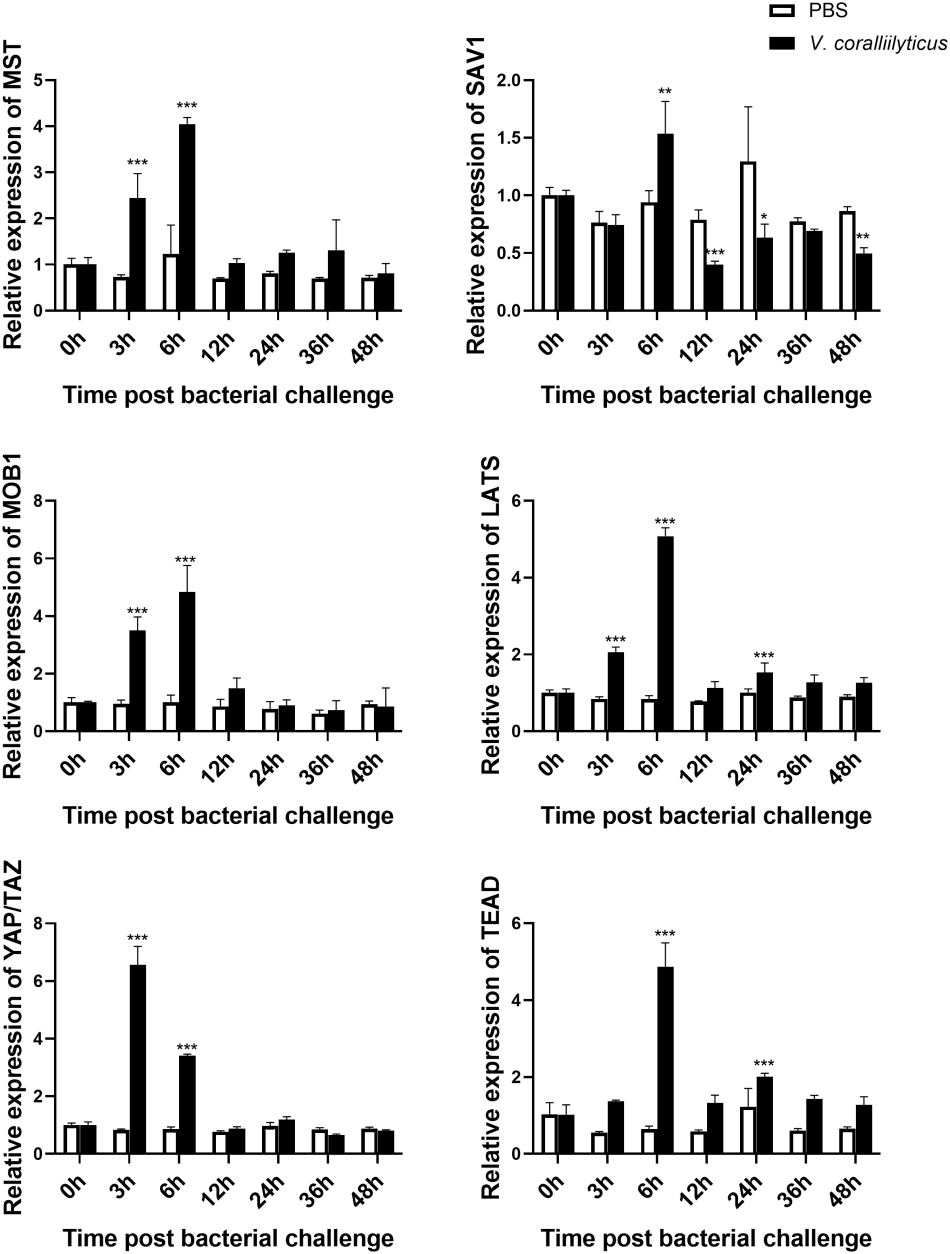
Expression profile of the Hippo pathway in *C. hongkongensis* post-challenge. The mRNA expression of core components of the Hippo pathway upon *V. coralliilyticus* challenge were examined by qRT-PCR. Data analysis was performed by using GraphPad Prism 9 software, and vertical bars represent mean ± SD (*n* = 3). ***p < 0.001; **p < 0.01; *p < 0.05.

To explore the potential function of Mst1/2, SAV1, Mob1A/B, Lats1/2, YAP/TAZ, and TEAD genes in oysters, we examined the mRNA expression levels by qRT-PCR in different tissues. As shown in **Figure 3**, all of these genes were expressed in various tissues, including the heart, hemocytes, mantle, gill, adductor muscle, digestive glands, and gonads. A relatively predominant expression level was observed in hemocytes, the major immune effecter cells, suggesting conserved involvement of the Hippo pathway in oyster defense against pathogens. Subsequently, we assessed the mRNA levels of these genes after infection with *V. coralliilyticus* (**Figure 4**). Interestingly, the expression levels of all six core members of the Hippo signaling pathway increased significantly in 6 h post-bacterial challenge. Some of these genes, including MST, MOB1, LATS, and YAP/TAZ, were induced as early as 3 h post-challenge, suggestive of a rapid response of the Hippo pathway to bacterial challenge.

### Crosstalk between the Hippo pathway and other signaling pathways in *C. hongkongensis*

**Figure 5:**
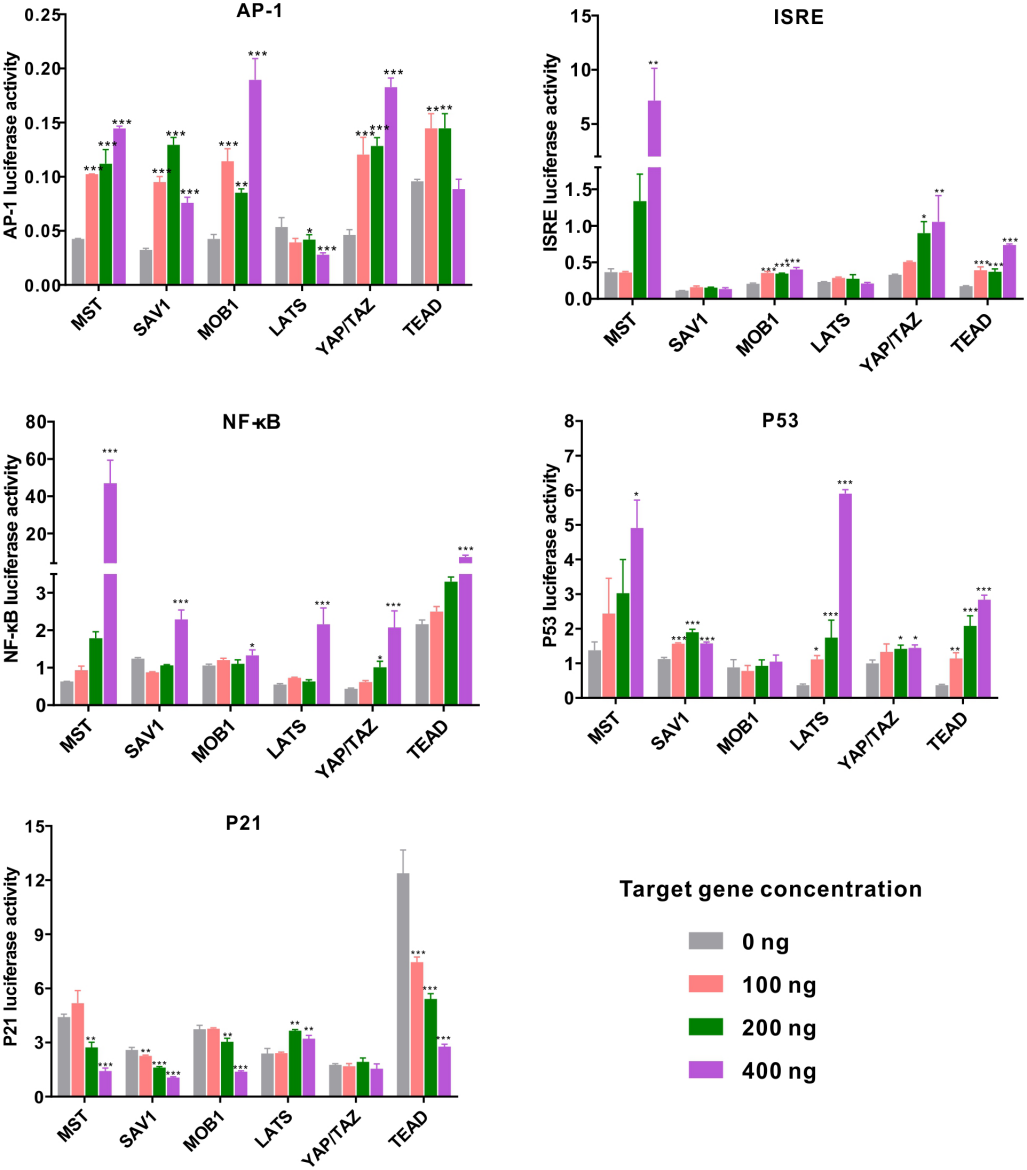
Relative luciferase activity in the eukaryotic expression system in HEK293T cells. Changes in the relative luciferase activity of AP-1, ISRE, NF-κB, p53, and p21 were analyzed after overexpression of *Ch*MST, *Ch*SAV1, *Ch*MOB1, *Ch*LATS, *Ch*YAP/TAZ, and *Ch*TEAD. Data analysis was performed by using GraphPad Prism 9 software, and vertical bars represent mean ± SD (*n* = 3). ***p < 0.001; **p < 0.01; *p < 0.05.

Previous studies have demonstrated complexity of the Hippo pathway and its crosstalk with various signaling pathways in innate immunity (20). In this study, we ventured to investigate the regulatory roles of *Ch*MST, *Ch*SAV1, *Ch*MOB1, *Ch*LATS, *Ch*YAP/TAZ, and *Ch*TEAD in different signaling pathways by using dual-luciferase reporter assays. Our results (**Figure 5**) show that these genes are significantly involved in multiple signaling pathways. Overexpression of *Ch*MST, *Ch*SAV1, *Ch*MOB1, *Ch*YAP/TAZ, and *Ch*TEAD significantly activated the AP-1 reporter, with peak increases of 3.40 folds, 2.35 folds, 4.45 folds, 3.95 folds, and 1.63 folds, respectively. However, *Ch*LATS slightly inhibited the luciferase activity of the AP-1 reporter at high concentrations. The ISRE reporter was significantly activated by *Ch*MST, with a luciferase activity increase of approximately 19.56 folds at high concentrations. Co-transfection of *Ch*MST and NF-κB reporter also elicited significant activation of NF-κB. Moreover, NF-κB was activated by other genes of the Hippo pathway as well.

Next, we examined the transcriptional activity of oyster Hippo pathway in p53 reporter genes. *Ch*MST, *Ch*SAV1, *Ch*LATS, *Ch*YAP/TAZ, and *Ch*TEAD enhanced the activity of p53 in a dose-dependent manner, with peak increases of around 3.56 folds, 1.5 folds, 16.0 folds, 1.4 folds, and 7.67 folds over control levels, respectively. The gene p21, an inhibitor in the p53-dependent apoptosis pathway, was significantly impaired by transfection with most genes of the Hippo pathway (*Ch*MST, *Ch*SAV1, *Ch*MOB1, and *Ch*TEAD), while no significant impact was observed in the *Ch*YAP/TAZ group. Overall, *Ch*MST appeared to be extensively involved in all pathways studied, suggesting a crucial role in innate immune response and apoptosis. Furthermore, all components of the Hippo pathway in *C. hongkongensis* activated the AP-1 reporter, and *Ch*MST and *Ch*TEAD were found to functionally implicate in ISRE, p53-p21, and NF-κB signaling pathways.

### Effects of MST and TEAD gene silencing on hemocyte apoptosis in *C. hongkongensis*

**Figure 6:**
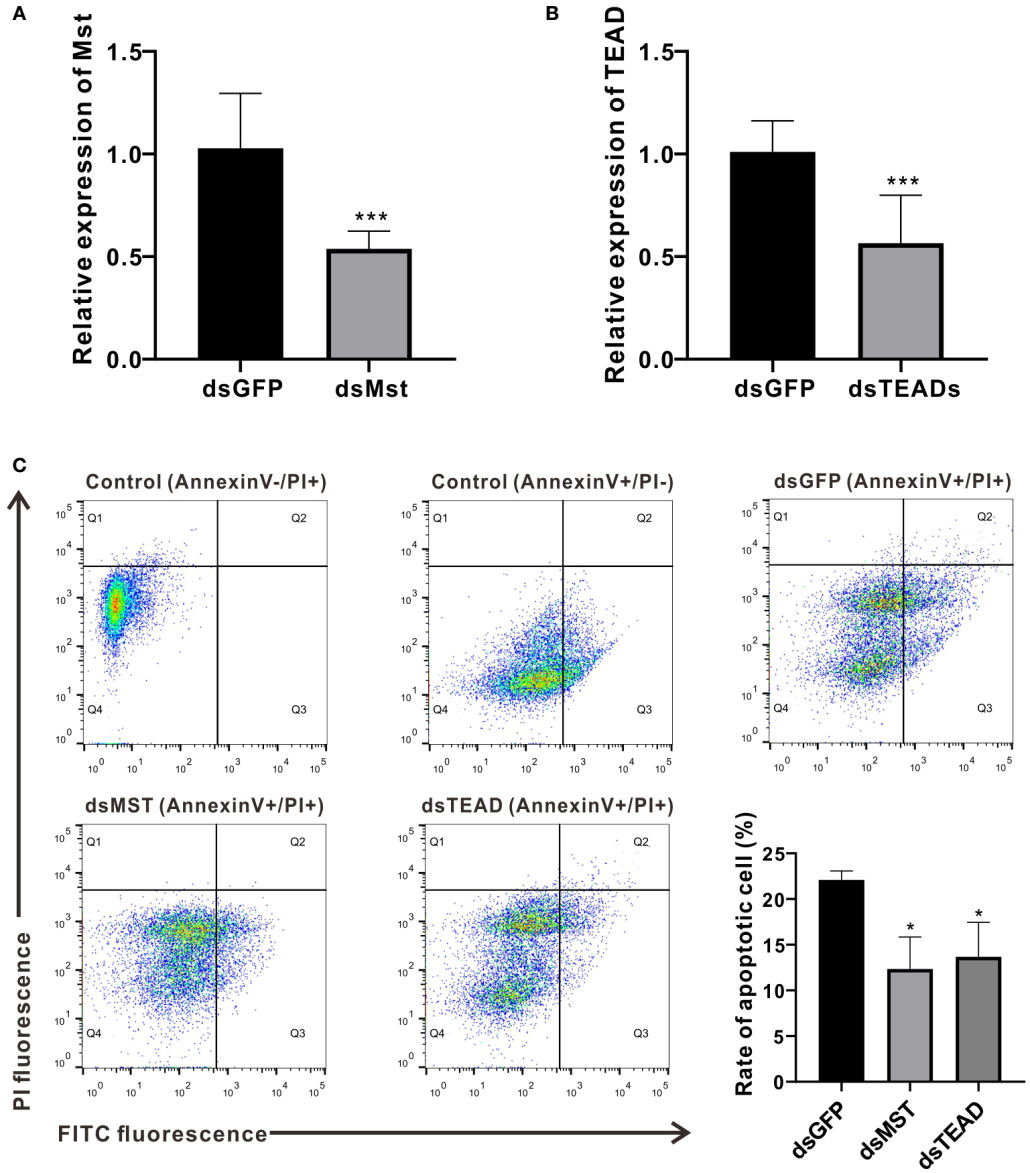
MST and TEAD gene participate in apoptosis. **(A)** Expression levels of *Ch*MST after RNA interference. **(B)** Expression of *Ch*TEAD after RNA interference. **(C)** Flow cytometry was used to detect hemocytes apoptosis after knockdown of *Ch*MST and *Ch*TEAD. Q1: Cells that were stained positively with PI only (upper left quadrant) were necrotic/non-viable cells. Q2: Cells that were stained double-positively by Annexin V-FITC and PI (top right quadrant) were cells undergoing late apoptosis/necrosis. Q3: On the lower right quadrant were cells stained positively by Annexin V only, representing cells undergoing early apoptosis. Q4: The lower left quadrant (PI and Annexin V negative cells) shows live cell populations. Lower right panel displayed the rate of apoptosis cells after *Ch*MST and *Ch*TEAD knockdown. Data analysis was performed by using GraphPad Prism 9 software, and vertical bars represent mean ± SD (*n* = 3). ***p < 0.001; *p < 0.05.

As MST and TEAD are major components of the Hippo pathway and exert prominent effects on crosstalk with the p53-p21 signaling pathway, we further investigated their roles *in vivo* by silencing their expression *via* dsRNA-mediated interference. Results from quantitative PCR reveal that the relative expression levels of MST and TEAD were reduced to approximately 52.31% and 55.99%, respectively, compared to the dsGFP control group (**Figures 6A, B**). To clarify their function in hemocyte apoptosis, we measured hemocyte apoptosis rates (including early and late apoptotic cells) in the dsMst and dsTEADs groups, which were approximately reduced from 22% to 12% and 14%, respectively, compared with rates of the dsGFP group (**Figure 6C**). These observations thus confirm that *Ch*MST and *Ch*TEAD are functionally conserved in the induction of cell apoptosis (21).

### Responses of several key immune-related genes to MST and TEAD gene silencing

**Figure 7:**
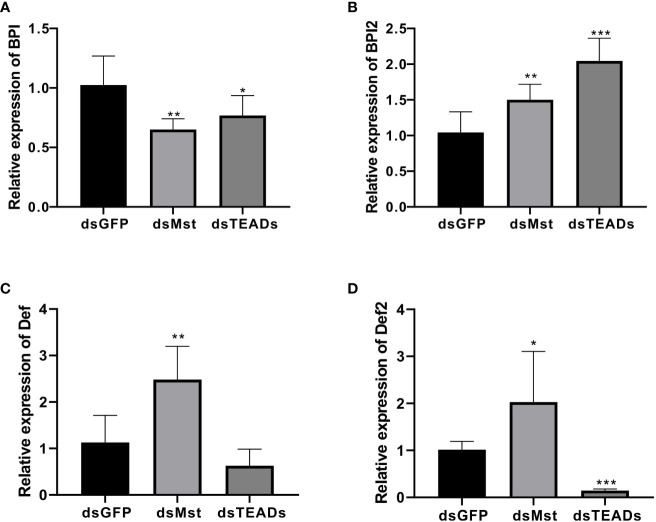
Responses of four immune-related genes to MST and TEAD gene silencing. The relative expression of BPI **(A)**, BPI2 **(B)**, Def **(C)**, and Def2 **(D)** in dsGFP, dsMst and dsTEADs groups was examined by qRT-PCR. Data analysis was performed by using GraphPad Prism 9 software, and vertical bars represent the mean ± SD (*n* = 3). ***p < 0.001; **p < 0.01; *p < 0.05.

In order to elucidate functional relevance of the Hippo pathway in immunological contexts, we measured the expression levels of several key immune-related genes (Def, Def2, BPI, and BPI2) following *Ch*MST and *Ch*TEAD interference. Our results show that depletion of *Ch*MST and *Ch*TEAD significantly decreased the expression of the BPI gene, while accelerating the expression of BPI2 *in vivo* compared to the GFP-dsRNA injection group (**Figures 7A, B**). As for Def and Def2, *Ch*MST and *Ch*TEAD had opposite effects. Impairment of *Ch*MST significantly increased the expression of Def and Def2 genes (**Figures 7C, D**), while these two genes were inhibited after *Ch*TEAD-dsRNA interference. These results strongly support the notion that the *Ch*MST and *Ch*TEAD-dependent Hippo pathway likely plays an important role in antimicrobial processes.

### Mst-mediated non-canonical pathway reduced hemocytes phagocytosis and bactericidal in *C. hongkongensis*

**Figure 8:**
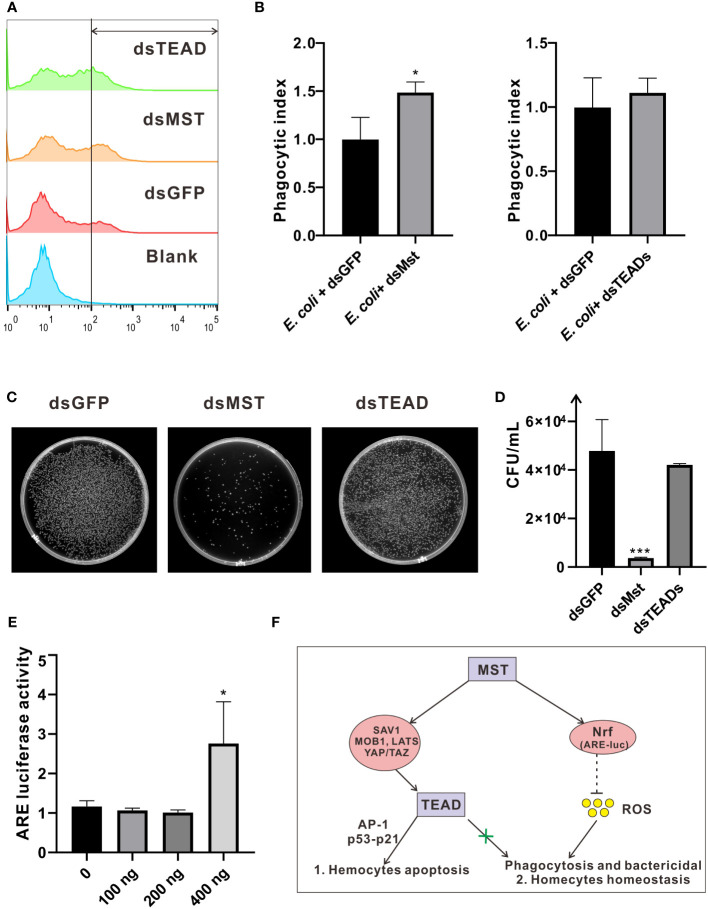
Roles of *Ch*MST in regulating hemocytic phagocytosis and bactericidal activity. **(A)** Hemocytic phagocytosis was detected by flow cytometry. Phagocytosis rates for *E coli and the* blank (PBS) control group were calculated and compared. **(B)** Phagocytic index was calculated to represent the effects of *Ch*MST (left panel) and *Ch*TEAD (right panel) in phagocytosis. Data analysis was performed by using GraphPad Prism 9 software, and vertical bars represent mean ± SD (*n* = 3). *p < 0.05. **(C)** Representative images for outcomes of bactericidal activity following an infection assay in hemocytes, with genetic knockdown of *Ch*MST or *Ch*TEAD. **(D)** Bactericidal survival rates were calculated by using Image J and presented with GraphPad Prism 9. Data were analyzed by unpaired *t*-test and presented as mean ± SD. ∗∗∗*p* < 0.001, *n* = 3. **(E)** Relative luciferase activity of ARE after overexpression of *Ch*MST. Data analysis was performed by using GraphPad Prism 9 software, and vertical bars represent mean ± SD (*n* = 3). *p < 0.05. **(F)** Conceptual framework of Hippo signaling in *C hongkongensis*.

In innate immunity, the role of MST as a core kinase of the Hippo signaling pathway is inherently important, as it is required in phagocytosis and efficient clearance of bacteria by sensing excessive ROS and modulating the activity of the key antioxidant transcription factor Nrf2 in a non-canonical manner. In this context, we investigated the function of MST in phagocytosis and bactericidal assays in oyster hemocytes. Specifically, injection of dsRNA of *Ch*MST significantly increased the rate of hemocyte phagocytosis of *E. coli* from approximately 20% to 30%, relative to the control group injected with dsGFP (**Figures 8A, B**). Moreover, compared with the control group, bacterial survival rate in the dsMst group starkly decreased, which was unaffected in the dsTEADs group (**Figures 8C, D**). However, *in vivo* interference of *Ch*TEAD had no influence on oyster hemocyte phagocytosis and bactericidal activity, suggesting participation of only *Ch*MST in the non-canonical Hippo pathway, but not other genes. To further clarify the mechanisms, we tested whether MST was functionally conserved in oyster by utilizing an ARE luciferase reporter. Dual-luciferase reporter assays showed that overexpression of *Ch*MST in mammalian cells significantly enhanced the activity of the ARE reporter at a high concentration of 400 ng (**Figure 8E**), supporting an essential role of *Ch*MST in Nrf-mediated signaling. Based on these observations, we proposed a framework of Hippo signaling in *C. hongkongensis* (**Figure 8F**).

## Discussion

As invertebrates, oysters have developed a highly sophisticated innate immune system, making them an attractive model for studying the evolution of host defense systems. Multiple classical signaling pathways have been associated with innate immunity in oysters, where they serve unique or conserved functions (21–23). Notably, the Hippo pathway is highly conserved in many organisms. It regulates cell apoptosis in a canonical manner, and phagocytosis in a non-canonical manner. In this present work, we showed that members of this signaling cascade are retained in *C. hongkongensis* and conserved in protein domains, while some have been lost across other species possibly due to genetic variation or substitution of their function by other genes. In accordance with prior research, it has been determined that the complete complement of Hippo pathway constituents remains conserved within bilaterians, including the cnidarian *Nematostella vectensis* (24). However, it has been observed that in the earlier species *Amphimedon queenslandica*, YAP displays a putative or degenerate homologous structure based on the findings. It can be assumed that the Hippo pathway evolved prior to the emergence of metazoans. Additionally, our findings demonstrate the existence of non-canonical signaling regulation, with species-specific features, at least in bivalves. However, the functions of non-canonical signaling pathways are less explored in lower organisms, and further research is required to ascertain their origins.

Furthermore, we have shown that these genes are highly expressed in oyster hemocytes, the principal immune effector cells located throughout connective tissues (25). Functionally, when a pathogenic microbe invades, the Hippo pathway responds immediately in oyster hemocytes, which promotes rapid killing of the microbe and prevention of disease. Although we did not explore the protein levels *in vivo*, an increase in transcriptional levels of these genes is similar to that of many reported immune genes, including those in the TLR-mediated signaling pathway, which is more commonly known (14, 26). Therefore, the Hippo signaling pathway is also relatively conserved in oyster function and is heavily involved in innate immune regulation, which is further supported by the results of signaling crosstalk, cell apoptosis, and phagocytosis analysis.

Signaling crosstalk is a recurring theme in Hippo pathway research. It has been primarily observed in immune systems involving Toll/IMD mediated anti-bacterial response in *Drosophila*, innate antiviral response, NF-κB signaling, and ROS production (20, 27). In the oyster, the Hippo pathway plays a similarly crucial role in coordinating cellular signaling, including that of the NF-κB, ISRE, AP-1, p53, and p21 pathways. Interestingly, AP-1 is frequently perceived as a pivotal transcription factor that determines cell survival or cell fate in response to extracellular stimuli (28), along with p53-dependent p21 signaling (29, 30). In the case of the oyster, the Hippo pathway significantly induced activities of AP-1 and p53 reporters, while the p21 reporter, which directs an anti-apoptotic response (30, 31), was conversely inhibited. Therefore, it is interesting to note that the Hippo pathway precisely regulates cell apoptosis, while *Ch*MST and *Ch*TEAD were further demonstrated to be functionally conserved and serve as proximal mediators of apoptosis in this study. Early on, we found that LHX9 induced cell apoptosis by activating apoptosis genes and signaling pathways (19). By combining the downregulation of the Hippo signaling pathway through LHX9 knockdown, as well as the promotion of apoptosis through Hippo pathway, we further demonstrated that the LHX9-Hippo signaling axis regulates the apoptosis of oyster hemocytes. However, subtle functional differentiation may exist among the immune effector proteins, as depletion of *Ch*MST and *Ch*TEAD resulted in differential expression profiles of defensin, suggesting the presence of specific, alternative regulatory mechanisms of *Ch*MST signaling in the oyster.

In general, the kinase MST is activated by Toll-like receptor signaling, or elevated ROS generation to enhance antimicrobial response (16). MST itself can sense ROS and contribute to cellular redox homeostasis by modulating stability of the antioxidant transcription factor Nrf2 (15). Interestingly, knockdown of *Ch*MST resulted in an increase in the phagocytosis rate and bactericidal ability of oyster hemocytes. Meanwhile, corroborating by evidence from dual-luciferase reporter assays on the ARE reporter, it suggests the existence of an MST-Nrf2 axis in the oyster. In addition, oyster hemocytes have a propensity to produce ROS even in the absence of stimulation (32, 33), which further lends support to our assumptions. ROS production invariably surges during bacterial infection. Therefore, we speculate that the Hippo pathway in the oyster can operate in a species-specific, non-classical manner, which involves principally reduction of ROS levels and maintenance of cellular homeostasis.

Collectively, our work here reveals that the oyster Hippo pathway is retained in a conserved, canonical regulatory manner, with features similar to reported model organisms. The pathway has crosstalk with many innate immune signaling pathways, mainly to mediate apoptosis. An interplay between ROS and the kinase MST also exists in oyster hemocytes, where MST functions as a brake on cellular ROS production to offer cytoprotective effects against oxidative injury. Overall, our findings confirm the conserved function of the Hippo pathway across evolutionary lineages, providing glimpses into species-specific regulatory mechanisms underlying cellular homeostasis under stress or normal conditions.

## Materials and methods

### Animals and sample collection

Healthy oysters (*Crassostrea hongkongensis*) were collected from Zhanjiang, Guangdong province, China, and were acclimated in aerated seawater (20% salinity) at 20°C for two weeks prior to the experiments. During the acclimation period, oysters were fed twice a day with the microalgae *Isochrysis galbana* (10^5^ cells/mL) and *Chaetoceros calcitrans* (2 × 10^5^ cells/mL). For tissue expression profile analysis, hemocytes, heart, adductor muscle, mantle, gonads, digestive glands, and gill tissues of nine healthy individual oysters (three per sample) were collected for RNA extraction. For the bacterial challenge experiment, 100 healthy oysters were randomly assigned to the experimental and control groups. Oysters in the experimental group were injected with 100 live bacteria *Vibrio coralliilyticus* (10^6^ CFU/mL suspended in phosphate-buffered saline, PBS) into the adductor muscles. Individuals in the control group were injected with an equivalent volume of sterile PBS. Hemocytes were collected at scheduled intervals (2, 4, 6, 12, 24, and 48 h after bacterial challenge) and centrifuged immediately. Five individuals were randomly sampled from each group at each specified time point post-injection.

### Identification of orthologs in the Hippo pathway

To search for orthologs of Hippo pathway genes, refseq protein data of 16 species were downloaded from NCBI, including *Homo sapiens*, *Danio rerio*, *Ciona intestinalis*, *Branchiostoma floridae*, *Acanthaster planci*, *Strongylocentrotus purpuratus*, *Bicyclus anynana*, *Drosophila melanogaster*, *Caenorhabditis elegans*, *Octopus bimaculoides*, *C. gigas*, *Mizuhopecten yessoensis*, *Nematostella vectensis*, *Acropora digitifera*, *Stylophora pistillata*, and *Amphimedon queenslandica*. Protein sequences of *C. hongkongensis* was obtained by whole-genome sequencing, which were published previously (34). Protein sequences of human and fruit fly hippo pathway genes were searched against refseq protein data for different organisms, representing different metazoan and non-metazoan groups. All blast hits were filtered with an e-value < 1.0E-20 and sequence length > 50. Subsequently, the hits were validated by means of a reciprocal blast search in NCBI, whereby best matches for the target genes was assigned as an ortholog.

### RNA isolation, cDNA synthesis, and quantitative RT-PCR analysis

Total RNA was isolated from collected samples by using TRIzol Reagent (Invitrogen, USA), following the manufacturer’s protocol. The quality and quantity of RNA were assessed by using a NanoDrop 2000C spectrophotometer (Thermo Fisher Scientific, USA). The RNA was then diluted to 1 μg/μL and used for cDNA synthesis with a PrimeScript RT reagent Kit (Takara, Japan). Gene-specific primers were designed by using the Primer-BLAST tool and synthesized by Sangon Biotech (Shanghai, China) to amplify a fragment of approximately 200 bp (**Table 1**). All qRT-PCR reactions were performed in triplicate by using SYBR Premix Ex Taq (Takara, Japan) on a CFX Connect Real-Time PCR Detection System (Bio-Rad, USA). Expression levels of the target genes were normalized to a reference gene, glyceraldehyde-3-phosphate dehydrogenase (GADPH). Relative expression of Hippo pathway genes was estimated by using the 2^−ΔΔCT^ method (35). The same methods were used to evaluate gene expression after RNA interference.

**Table 1:** Primers used in this study.

Primer name	Sequence (5’-3’)
qMST-F	ATTGTGGAGCTGGCTCAGTG
qMST-R	TCCGTGTTCAGCAGGATGTT
qSAV1-F	TGTTCCTGCCAACCCATACC
qSAV1-R	TTCTTCCGTCGCTCCATCTC
qLATS-F	GACAGCCTCAGTCATATTCG
qLATS-R	ACATTCCATCGTCGTTCC
qMOB1-F	CTTGCCCTACTGTGAAGAAA
qMOB1-R	AAGTGCTGGTGGTAAATGTG
qYAP/TAZ-F	GCAAGAGGCAAGAAGACCTA
qYAP/TAZ-R	CTCCTGCTTGATGTGATTGG
qTEAD-F	ACAAACCCGCGACCCAGATA
qTEAD-R	CGCCCAGAACTTGACAAGAAAG
qDef-F	AAAACATCAGCAATTAAATCC
qDef-R	AGTCATCCGTTAGTCGTACC
qDef2-F	GCAAATCAATCAACAACGCA
qDef2-R	GCAGATCCACAATCCACCAC
qBPI-F	AACACCACAGTGATGTTATACAT
qBPI-R	TAGCTGACAGCGTTAGGAAG
qBPI2-F	CTGGTTGTCCGACTTCCTCT
qBPI2-R	TCCGTCTGCATCCACTCTTG
MST-F	ATGACTAGTCAAAGTGACCCCCTAA
MST-R	GAAATTGTTCTGTCTTTTCTTCTTG
SAV1-F	ATGTTATCGAAGAAAAAAGACTCTG
SAV1-R	AACTTTGGTTTCAATGTTCTGTGTA
LATS-F	ATGATTTCCCGAAAAGAGGACCCCA
LATS-R	GACATACACCGGTGCACTGGAGGAG
MOB1-F	ATGAGCTTTCTGTTTGGAGGCCGAA
MOB1-R	CTAGCGATCCTTGCTGGTCAGCTTA
YAP/TAZ-F	ATGTCGCAGGACATGCAAGAACGGA
YAP/TAZ-R	CAGCCAAGTAAGTGAATTGTCCCCA
TEAD-F	ATGTCTTCAGCGTGGAACAGCGAGA
TEAD-R	GTCTTTCACCAGTCTGTAGATGTGG
dsMst-F	GGATCCTAATACGACTCACTATAGGACAGAGGAGGAGATTGCTACG
dsMst-R	GGATCCTAATACGACTCACTATAGGGACCATTTATCGGGGTTTCGG
dsTEAD-F	GGATCCTAATACGACTCACTATAGGGGGTGTGGAGCCCAGACATTGAAC
dsTEAD-R	GGATCCTAATACGACTCACTATAGGCCCAGGGTAGGAGGGCCTGACATT
dsGFP-F	GGATCCTAATACGACTCACTATAGGAGCAGAACACCCCCATCGG
dsGFP-R	GGATCCTAATACGACTCACTATAGGAGTTTGGACAAACCACAAC
GADPH-F	GGATTGGCGTGGTGGTAGAG
GADPH-R	GTATGATGCCCCTTTGTTGAGTC

### Plasmid construction, cell culture, transient transfection, and dual-luciferase reporter assays

The ORF cDNA fragments of *Ch*MST, *Ch*SAV1, *Ch*MOB1, *Ch*LATS, *Ch*YAP/TAZ, and *Ch*TEAD were cloned into a pcDNA3.1 expression vector by homologous recombination by using a Vazyme™ One Step Cloning Kit. Human embryonic kidney 293T (HEK293T) cells were used to perform the dual-luciferase reporter assay. The cells were cultured in Dulbecco’s modified Eagle medium (DMEM, Gibco, USA), supplemented with 10% heat-inactivated fetal bovine serum (FBS, Gibco, USA) and antibiotics (100 mg/L streptomycin and 10^5^ U/L penicillin; Gibco) under 5% CO_2 at_ 37 °C in a humidified atmosphere. Prior to transfection, HEK293T cells were seeded onto 48-well plates and cultured for 24 h. Lipofectamine 8000™ transfection reagent (Beyotime, Shanghai, China) was used to transfect the cells with plasmid DNA, according to the manufacturer’s protocol. For dual-luciferase reporter assays, HEK293T cells were co-transfected with a pRL-TK vector (20 ng/well), reporter genes (AP-1-Luc, P53-Luc, ISRE-Luc, NF-κB-Luc, P21-Luc, and ARE-Luc) (200 ng/well), expression vectors with the target gene (0, 100, 200, and 400 ng/well), or empty expression vectors (400, 200, 100, 0 ng/well).

After 48 h of transfection, the cells were washed with PBS and collected for subsequent assessment in the Dual-Luciferase Reporter Assay (Promega). Relative luciferase activity of each trial was calculated as firefly luciferase activity relative to Renilla luciferase activity. Each transfection was performed in quintuplicate, and each experiment was repeated three times.

### RNA interference

cDNA fragments of *Ch*MST, *Ch*TEAD, and a GFP cDNA fragment (negative control) were amplified with primers in conjunction with T7 promotor sequences (**Table 1**) and utilized as templates to synthesize the dsRNA according to the manufacturer’s instructions of the T7 RiboMAX™ Express RNAi System (Promega, USA). Thirty oysters were randomly assigned into three groups and placed in three tanks: the dsMst, dsTEAD, and dsGFP groups. Each oyster was injected with 100 μg dsRNA. Three days after injection, hemocytes from five individuals were collected to form biological replicates. These samples were immediately centrifuged (300 g/min for 5 min at 4°C) to harvest hemocytes, followed by RNA extraction and subsequent analyses.

### Flow cytometric analysis

An apoptosis detection kit (Vazyme, China) was used in flow cytometric analysis, according to the manufacturer’s instructions. Hemocytes were collected by centrifugation at 300× g for 5 min at 4°C and washed twice with precooled PBS. Approximately 10^5^ hemocytes were resuspended in 100 μL of 1× binding buffer and incubated with 5 μL Annexin V-FITC and 5 μL propidium iodide (PI) for 10 min at room temperature in the dark. A cell suspension was then supplemented with another 400 μL of 1× binding buffer. Cell apoptosis was detected by gating at least 10,000 cell events by using Guava easyCyte, followed by analysis by using the FlowJo software.

### Phagocytosis assay

*Escherichia coli* (strain DH5*α*) was cultured at 37°C for 12 h in Luria-Bertani (LB) medium and transformed with a pFPV25.1 plasmid for green fluorescence emission. The bacteria were then centrifuged at 800× g for 10 min at 4°C and washed three times in PBS. Bacterial cell density was adjusted to OD_600nm_ = 1.0 in PBS, and bacterial cells were added to the hemocyte culture in a 12-well plate at a ratio of 50:1 for 15 min. Hemocytes were washed three times with Tris buffer (pH 8.0, 50 mM), and Trypan blue was added to inhibit further attachment of bacteria to hemocytes (36). A cell suspension was then supplemented with PBS containing 1.5‰ EDTA. Phagocytosis-related fluorescence in oyster hemocytes was quantified by a flow cytometry assay. Phagocytosis was monitored by gating at least 10,000 events per sample, whose data were analyzed by using the FlowJo software.

### Bacterial clearance assay

*E. coli* (strain DH5*α*) was cultured at 37°C until OD_600nm_ = 0.2, harvested by centrifugation at low speed, and washed three times with Tris buffer (50 mM, pH 8.0; A610195, Sangon Biotech). Approximately 2.5× 10^5^ hemocytes per well were seeded into a 24-well plate and challenged with *V. parahaemolyticus* at an MOI of 50 at room temperature for 30 min. Cells were then briefly treated with 0.02% trypsin-EDTA four times to remove extracellular bacteria and subsequently lysed in 1 mL PBS containing 0.05% Triton X-100. Finally, 50 μL of the lysate was inoculated on LB agar plates to enumerate bacterial colonies. All tests were performed in triplicate and repeated three times.

### Statistical analysis

Statistical analyses were performed by using GraphPad Prism (version 9.0.0). All statistical values were expressed as mean ± SD. Statistical significance between groups was determined by Student’s *t*-test, while comparisons for more than two groups were done by one-way ANOVA or two-way ANOVA relative to the control group, and indicated by asterisks (*p < 0.05, **p < 0.01 and ***p < 0.001).

## Data availability statement

The original contributions presented in the study are included in the article/supplementary material. Further inquiries can be directed to the corresponding authors.

## Author contributions

FM, ZY, and YZ contributed to conception and design of the study. FM, XZ, ZY, N-KW, and WY researched data and contributed to the discussion. XZ, FM, and SF performed the statistical analysis XZ and FM wrote the first draft of the manuscript. SX and JS prepared oyster for experiment. ZX and SF cloned the genes. N-KW and YB contributed to manuscript revision. All authors contributed to the article and approved the submitted version.
